# The Effect of Sleep Habits on Quality of Life in Pediatric Patients With Chronic Kidney Disease

**DOI:** 10.7759/cureus.64585

**Published:** 2024-07-15

**Authors:** Sevgin Taner, Gunay Ekberli, Serkan Gunes

**Affiliations:** 1 Pediatric Nephrology, Adana City Training and Research Hospital, Adana, TUR; 2 Pediatric Urology, Adana City Training and Research Hospital, Adana, TUR; 3 Child Psychiatry, Adana City Training and Research Hospital, Adana, TUR

**Keywords:** children, kidney replacement therapy, quality of life, sleep disorder, chronic kidney disease

## Abstract

Background: Sleep disturbance has been studied in adult patients with early and end-stage chronic kidney disease (CKD). However, there are limited publications on the pediatric patient population. This paper evaluated the association between sleep disturbances and quality of life (QoL) in pediatric patients with CKD.

Methods: The study included 22 patients and 22 healthy controls from the pediatric nephrology outpatient clinic. All participants completed the Turkish Generic Health-Related Quality of Life Questionnaire for Children and Adolescents (HRQoLQ) and the Child Sleep Habits Questionnaire (CSHQ). Patients diagnosed with CKD were compared in terms of HRQoLQ and CSHQ scores within themselves as kidney replacement therapy (KRT) recipients and non-recipients and with the control group.

Results: The mean HRQoLQ total score of the patients was 89.0 ± 12.4 and the mean CSHQ total score was 46.7 ± 5.6; there was no correlation between the total scores (p=0.599). CSHQ total and subgroup scores were similar in patients with and without KRT. The CSHQ total and subgroup median scores were not different in the patient and control groups. According to the HRQoL scale, the total QoL score and the physical and emotional well-being subscale scores were lower in patients receiving KRT than in those not receiving KRT.

Conclusion: Sleep problems and HRQoL should not be underestimated in the pediatric CKD population, especially in patients receiving KRT. Large-scale studies with long-term outcomes are needed to understand better and improve QoL.

## Introduction

Chronic kidney disease (CKD), associated with irreversible damage to kidneys that can progress to kidney failure also known as end-stage renal disease (ESRD), is defined as kidney damage for ≥3 months or a glomerular filtration rate (GFR) of less than 60 mL/min/1.73 m^2^ for ≥3 months. Patients with ESRD are commenced on kidney replacement therapy (KRT) such as hemodialysis (HD), peritoneal dialysis (PD), or kidney transplantation [1]. ESRD has devastating consequences with a 30-fold increased risk of mortality and is characterized by specific problems and morbidity risks, such as cardiovascular problems, growth impairment, and psychosocial adjustment, which severely affect the quality of life (QoL) in children [2]. Approximately 40-85% of patients on dialysis reported to have poor sleep patterns. Sleep disturbances are common in both early and ESRD [3-6].

The etiology of sleep disturbance in patients with CKD is reported to be multifactorial. The aforementioned factors can be listed as demographic factors, habits, biological parameters, medical comorbidity, treatment-related factors, and psychosocial conditions. Poor sleep quality has been found to be associated with morbidity and mortality both in the pre-dialysis period and in CKD patients receiving HD [7,8]. Based on our clinical practice, it can be claimed that sleep disturbances are one of the most important factors affecting the QoL and treatment compliance of patients with CKD. The aim of this study was to evaluate the frequency of sleep disturbances in pediatric patients with CKD compared to the healthy control group and the effects of these disturbances on QoL. We also aimed to evaluate the effect of CRT on sleep and QoL in pediatric patients with CKD. For this purpose, the scores of the scale evaluating sleep habits and QoL completed by pediatric patients diagnosed with CKD and a healthy control group were compared.

## Materials and methods

Study design

This is a case-control study, evaluating the effect of sleep disturbance on QoL in children with CKD. Patients who were followed up at the pediatric nephrology and urology outpatient clinics of Adana City Training and Research Hospital and who agreed to fill in the questionnaire were included in the study. Adana City Training and Research Hospital is a large regional hospital located in the south of Turkey, close to the Syrian border.

The study population was selected from patients with CKD (stages 3-5). The control group was selected among patients who applied to the outpatient clinic and were determined to be healthy. The definition and classification of CKD were evaluated according to Kidney Disease: Improving Global Outcomes guidelines [9]. Accordingly, patients with a GFR of 45-59 mL/min/1.73 m^2^ were evaluated as Grade 3 CKD, 15-29 mL/min/1.73 m^2^ as Grade 4 CKD, and <15 mL/min/1.73 m^2^ was referred to as Grade 5 CKD or ESRD. HD and PD were commenced on the patients with ESRD as KRT.

After informed consent had been provided by all participants’ parents, they were requested to complete The Turkish Generic Health-Related Quality of Life Questionnaire for Adolescents (HRQoL) and The Child Sleep Habits Questionnaire (CSHQ), which were validated in Turkish pediatric patients with CKD.

Inclusion and exclusion criteria

The study included pediatric patients 6-18 years of age who are being treated with a diagnosis of CKD. In the selection of patients diagnosed with CKD, the inclusion criteria were an absence of any mental disabilities or mobilization problems. All children were mobile either with a wheelchair or spontaneously. Patients with acute illness (e. g. infections, clinical instabilities) were not scheduled for interviews. Exclusion criteria were patients <6 years old, patients who do not want to fill in the scales, illiterate patients, and patients diagnosed with mental retardation (having an IQ below 70).

Controls

The control group consisted of healthy sex- and age-matched children who applied to the outpatient clinic and were determined to be healthy. No medical or family history of renal diseases was detected in the control group. Healthy status was determined through a review of the medical history and either a parental report or self-report to rule out the presence of chronic or acute diseases.

Clinical and laboratory measurements

Clinical characteristics, anthropometric measurements, and laboratory and radiological tests were evaluated during the clinic visit and by reviewing medical records at the time of the interview. Clinical data included gender, age, height, weight, primary etiology of CKD, stage of CKD, treatment compliance, and received kidney replacement treatments. Laboratory tests included serum levels of blood urea nitrogen (BUN), creatinine, and hemoglobin levels.

Tools

Sociodemographic Characteristics Data Form

The authors created a questionnaire including information on sociodemographic features of children and caregivers. This form contains information such as the child's name, surname, age, sex, number of siblings, level of education, and family history. The form applied to all caregivers and children.

The Turkish Generic Health-Related Quality of Life Questionnaire for Children and Adolescents (HRQoLQ)

The HRQoLQ was developed by Ravens Sieberer and Bullinger in 1998 [10]. Eser et al. published the Turkish version of the scale in 2004 [11]. This scale consists of 24 items on a 5-point Likert scale (from 1 = “never” to 5 = “always”). The questionnaire includes six subscales: physical health, emotional health, self-esteem, family, friends, and school. Raw scores are converted to a scale of 0-100. Higher scores indicate better HRQoL.

The CSHQ

The CSHQ is a parent-report sleep screening instrument consisting of a 33-item questionnaire. It consists of the total score and subscales. Subscales include bedtime resistance (six items), sleep onset latency (one item), sleep duration (three items), sleep anxiety (four items), night awakenings (three items), parasomnias (seven items), sleep-disordered breathing (three items), and daytime sleepiness (eight items). Three additional questions collect information about evening bedtime, morning wake-up time, and total sleep time. Parents are asked to report their child's average sleep behavior over the past week. Items are rated on a 3-point scale of often (5 to 7 times per week), sometimes (2 to 4 times per week), and rarely (0 to 1 time per week). A higher CSHQ score indicates more sleep problems [12]. 

Ethical considerations

Ethics committee approval was obtained from the Local Ethics Committee of Adana City Training and Research Hospital for the study (Approval Date: 03/08/2023; Approval Number: 2756).

Statistical analysis

Statistical analysis was done using SPSS, version 25 (IBM Corp., Armonk NY). The normality assumption of quantitative data was assessed in each group by the Shapiro-Wilk test. Descriptive data are shown as percentages, mean ± standard deviation (SD) for normally distributed data, and median (range) for non-normally distributed data. Pearson's chi-square test and Fisher’s exact test were used for comparison of categorical variables. For analyzing independent continuous variables, Student's t-test was used under parametric conditions and the Mann-Whitney U test under nonparametric conditions. A p-value <0.05 was considered statistically significant.

## Results

The study population consists of 22 patients (12 male/10 female) and 22 controls (11 male/11 female). The median age of the patients was 14 (8-16) years and that of the controls was 12 (8-16) years. Groups were similar in terms of age and sex (p=0.127, p=0.763). Considering the primary diagnoses causing CKD in the patient group, congenital anomalies of the kidney and urinary tract were the leading cause in 11 patients (50.0%). Other causes were tubular diseases in four (18.2%) patients, glomerular diseases in three (13.6%), inherited renal disorders in two (9.1%), ciliopathy in one (4.5%), and one (4.5%) with unknown etiology. The median time spent with the diagnosis of chronic CKD was 3.8 years (1.8-15.7). The distribution of the patients according to CKD stages was as follows: 14 (63.6%) were being followed up with stage 5 CKD, five (22.7%) with stage 4 CKD, and three (13.6%) with stage 3 CKD. Of the 13 patients diagnosed with stage 5 CKD, 12 were on HD and two were on PD as KRT. Patients’ characteristics are shown in Table 1.

**Table 1:** Patients’ characteristics CKD: chronic kidney disease. *Median (min-max).

Patients' Characteristics	n (%), N=22
Gender Distribution of the Patients (Male/Female)	12/10
Age (years)*	14 (8-16)
Time Since Diagnosed With CKD (years)*	3.8 (1.8-15.7)
CKD Etiology	
Congenital Anomalies of the Kidney and Urinary Tract	11 (50%)
Tubular Disease	4 (18.2%)
Glomerular Disease	3 (13.6%)
Inherited Renal Disorder	2 (9.1%)
Ciliopathy	1 (4.5%)
Unknown	1 (4.5%)
CKD Stages	
CKD Stage 3	3 (13.6%)
CKD Stage 4	5 (22.7%)
CKD Stage 5	14 (63.6%)
Kidney Replacement Therapy	N=14
Hemodialysis	12 (14.2%)
Peritoneal Dialysis	2 (85.7%)

The mean HRQoL total score of the patients was 89.0±12.4 and the mean CSHQ total score was 46.7±5.6. There was no correlation between the total scores (p=0.599). Patients were grouped as those who received KRT and those who did not. The mean CSHQ total score was 46.6±6.0 in patients with KRT and 46.9±6.0 in patients without. There was no difference between groups. CSHQ subgroup scores were also similar in patients with and without KRT. The mean HRQoL total score was lower in patients with KRT than those without (84.7±11.4 vs 97.2±10.5). According to the HRQoLQ, physical and emotional well-being subscale scores of patients receiving KRT were found to be lower than those who did not receive KRT. The disease subgroup scores of children who received KRT were also lower than those who did not. The comparison of the scale results of children who received and did not receive KRT is shown in Table 2. Total QoL score and illness, physical, and emotional well-being subscale scores were not correlated with the duration of CKD, respectively (p=0.766, p=0.647, p=0.667, p=0.578).

**Table 2:** Comparison of Health-Related Quality of Life Questionnaire and The Child Sleep Habits Questionnaire scores of the patients with and without renal replacement therapy *Independent-samples t test; **Mann-Whitney U test.

	Kidney Replacement Therapy (-)		Kidney Replacement Therapy (+)		p-Value*
The Child Sleep Habits Questionnaire	Mean±SD	Median (Min-Max)	Mean±SD	Median (Min-Max)	
Total Score	46.9±6.0	45 (40-57)	46.6±6.0	46 (39-58)	0.906
Bedtime Resistance	10.0±2.4	10 (7-13)	9.4±2.3	10 (6-14)	0.584
Sleep Duration	3.6±0.7	4 (3-5)	4.8±1.8	4 (3-8)	0.238**
Sleep Anxiety	6.9±2.2	7 (4-10)	6.4±2.2	6 (4-10)	0.664**
Night Wakings	5.1±1.1	5 (3-7)	4.3±1.3	5 (3-7)	0.238**
Parasomnias	8.6±1.8	8 (7-11)	8.6±2.3	8 (7-13)	0.815**
Sleep-Disordered Breathing	3.4±0.7	3 (3-5)	3.3±0.8	3 (2-5)	0.973**
Daytime Sleepiness	12.0±2.2	13 (8-14)	12.9±2.3	13 (8-16)	0.401
Health-Related Quality of Life Questionnaire for Adolescents	Mean± SD	Median (Min-Max)	Mean± SD	Median (Min-Max)	p-Value*
Total Score	97.2±10.5	97 (86-114)	84.7±11.4	79 (70-98)	0.029**
Physical Well-being	16.5±3.5	16 (12-22)	12.1±3.4	12 (4-17)	0.009
Emotional Well-being	17.0±2.4	17 (13-20)	14.0±2.6	15 (9-17)	0.013**
Self-esteem	15.6±28	16 (12-20)	14.3±4.4	15 (8-20)	0.440
Family	18.5±1.7	19 (16-20)	17.3±3.4	19 (8-20)	0.402**
Friends	15.8±2.7	16 (13-20)	14.4±3.2	15 (5-18)	0.285
School	13.7±2.3	13 (11-18)	12.4±3.9	12 (6-19)	0.382
İllness	20.8±4.9	21 (15-30)	16.6±2.6	16 (12-20)	0.019

**Figure 1:**
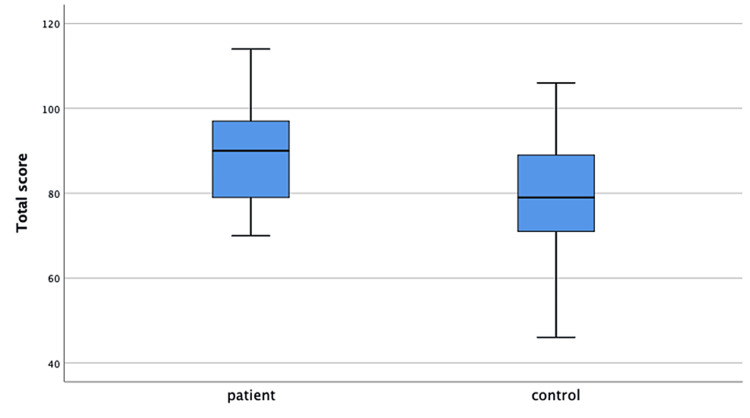
Health-Related Quality of Life Questionnaire total scores of the patient and control groups

**Figure 2:**
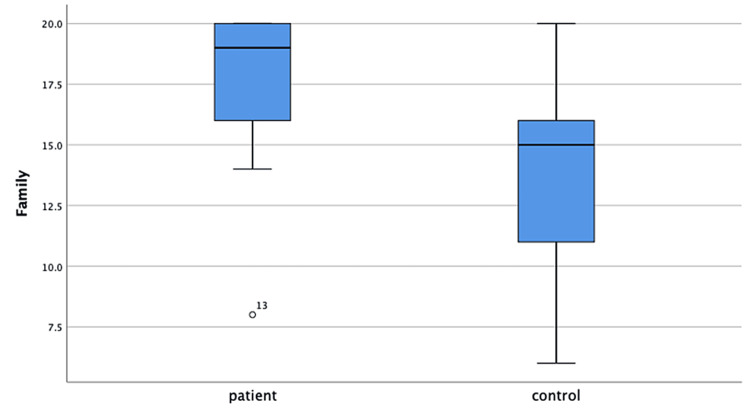
Health-Related Quality of Life Questionnaire family subgroup scores of the patient and control groups

The median CSHQ total score of the patients was 45.5 (39-58) and of the controls was 49.0 (29-66). The CSHQ total and subgroup median scores were not different in the patient and control groups. Patient and control group median scores are shown in Table 3. In the comparison of HRQoL scale scores, the mean total score of the patients was 89.0±12.4 and of the controls was 79.4±15.2. The patient group had a significantly higher total score than the control group (p=0.028). The patients’ mean family subgroup score was statistically significantly higher than that of controls (17.6±2.9 vs. 13.9±3.7, p=0.001) (Figures 1, 2). The scores of physical well-being, emotional well-being, self-esteem, social environment, and school subgroups were similar in both groups (Table 3).

**Table 3:** Comparison of Health-Related Quality of Life Questionnaire and The Child Sleep Habits Questionnaire scores of the patient and control groups *Mann-Whitney U test; **Independent-samples t-test.

The Child Sleep Habits Questionnaire	Patients' Median (min-max)	Controls' Median (min-max)	p-Value*
Total Score	45.5 (39-58)	49.0 (29-66)	0.488
Bedtime Resistance	9.5 (6-14)	8.0 (6-14)	0.079
Sleep Duration	4.0 (3-8)	4.5 (3-9)	0.370
Sleep Anxiety	6.0 (4-10)	6.0 (3-12)	0.417
Night Wakings	5.0 (3-7)	5.0 (3-9)	0.961
Parasomnias	8.0 (7-13)	9 (7-14)	0.451
Sleep-Disordered Breathing	3.0 (2-5)	3.0 (3-5)	0.520
Daytime Sleepiness	13 (8-16)	13 (8-18)	0.785
Health-Related Quality of Life Questionnaire for Adolescents	Patients' Mean (±SD)	Controls' Mean (±SD)	p-Value**
Total Score	89.0±12.4	79.4±15.2	0.028
Physical Well-being	13.7±3.9	14.5±3.8	0.672
Emotional Well-being	15.1±2.8	14.2±2.9	0.385
Self-esteem	14.8±3.8	13.5±4.0	0.270
Family	17.6±2.9	13.9±3.7	0.001
Friends	14.9±2.9	13.5±3.0	0.143
School	12.9±3.3	11.8±3.4	0.305

## Discussion

CKD develops as a result of progressive and irreversible damage to kidney tissue [13]. The diagnosis, management, and lifelong treatment of pediatric patients with CKD can cause not only physical but also social and psychological problems [14]. The social, behavioral, and psychological well-being of patients and caregivers is important for treatment compliance. The clinical manifestations of sleep disturbances in children may vary according to the age and developmental status of the child. It can affect physical, cognitive, emotional, and social development. All of these components affect the patient's ability to comply with treatment.

Although sleep disturbances are common in adults with CKD, there is limited information in the literature on the prevalence of sleep problems in children and adolescents [15]. The pathogenesis of this condition is not well understood and several factors have been associated with its occurrence, including chronic uremia, metabolic acidosis, iron deficiency, and hypertension [6]. A recent review evaluated updated information on the epidemiology, associated factors, and management of CKD-associated restless legs syndrome in both adult and pediatric populations. This update of the literature showed that restless legs syndrome is more common in patients with CKD than in the general population [16]. A quantitative meta-analysis to estimate the prevalence of sleep disorders in pediatric patients with CKD was performed by Kang KT et al. As a result, the prevalence rates of various sleep disorders were reported to be higher in children on dialysis [17]. CKD-associated pruritus is reported to be an under-recognized and under-treated cause of poor sleep quality, particularly in patients receiving KRT [18]. In our study, we could not document a significant difference between the patient/control group and the KRT+/KRT- group about the CSHQ total and subgroup median scores. The socio-culturally limited awareness of the importance of sleep in the life of the patient among the caregivers included in the study in the region where the study was conducted may explain the results, which are not consistent with the literature.

In a review of seven studies, the prevalence of sleep disturbances in the pediatric population was reported to be 77-85% in dialysis patients, 32-50% in transplant patients, and 40-50% in non-dialysis patients. They stated that an increased prevalence of sleep disturbance in children with CKD plays a critical role in HRQoL [6]. Another study by Davis et al. found that lower HRQoL scores were associated with sleep disturbances in pediatric patients with CKD [15]. Beak et al. in their study claimed that the HRQoL of CKD patients was poor and blamed factors were gender, GFR, socioeconomic status, presence of comorbidities, anemia, growth retardation, and behavioral disorders. Sleep disturbance wasn't mentioned as a contributory factor [19]. Another case-control study by Stabouli et al. aimed to assess the prevalence of sleep-related disturbances in children with CKD and to investigate possible correlations with measures of executive function. They found that children's behavioral regulation was not affected by sleep disturbances [20]. In our study, the lack of correlation between HRQoLQ and CSHQ can be explained as follows: as mentioned in the Results section, the study group also included patients under medical treatment (stage 3 and 4 CKD). Therefore, the patient/caregiver is not fully exposed to the difficulties of the process before receiving KRT. This may not be fully reflected in the psychology and also in the questionnaire scores. The small sample size may be another explanation for the lack of correlation.

Gerson et al. reported that even children with mild to moderate CKD, stages 1 to 3, had poorer overall HRQoL and worse physical, school, emotional, and social functioning compared to healthy children [21]. Consistent with the literature, overall HRQoLQ scores were lower in patients on KRT compared to those who weren't on KRT in our study. However, HRQoL was not associated with the duration of CKD. This may be due to constant factors that always affect the QoL of all pediatric patients, such as frequent hospital visits, long-term multiple drug treatments, long-term hospitalization, social isolation, and school absenteeism. The limited number and size of studies showing that sleep disturbances reduce QoL in these patients confirms the need for retrospective, controlled studies to better understand this problem and develop solutions [22]. Another surprising result is a positive advantage of the patient group compared to the control group on the HRQoLQ. The statistically significant difference in the scores of the family subgroup shows the importance of family support in overcoming difficulties and its impact on the QoL of CKD patients who face challenges in follow-up and treatment. The fact that CKD patients completed the scale more seriously than the control group may have influenced the results.

Limitations

The reason for the unexpected results in our study, such as higher QoL in the patient group compared with the control group and no significant difference in sleep disturbance, may be due to the small sample size, and the scales were mostly completed by caregivers, not patients. One of the main limitations of the study is the fact that the control group may not have taken the completion of the questionnaire very seriously. In this context, sleep studies such as polysomnography may provide more accurate results.

## Conclusions

Sleep disturbance should not be underestimated in pediatric patients with CKD. It is not always associated with QoL. In this study, we could not prove the presence of sleep disturbances and their negative impact on QoL in pediatric patients with CKD. Although the QoL scores of pediatric patients with CKD are better than the control group, especially with the high score in the family subscale, receiving KRT has a negative effect on QoL. Family support and social boundaries are important to overcome challenges during follow-up. Larger studies with long-term outcomes are needed to understand better and improve QoL.

## References

[1] Harada R, Hamasaki Y, Okuda Y, Hamada R, Ishikura K (2022). Epidemiology of pediatric chronic kidney disease/kidney failure: Learning from registries and cohort studies. Pediatr Nephrol.

[2] Harambat J, van Stralen KJ, Kim JJ, Tizard EJ (2012). Epidemiology of chronic kidney disease in children. Pediatr Nephrol.

[3] Tu CY, Chou YH, Lin YH, Huang WL (2019). Sleep and emotional disturbance in patients with non-dialysis chronic kidney disease. J Formos Med Assoc.

[4] De Santo RM, Bilancio G, Santoro D, Vecchi ML, Perna A, De Santo NG, Cirillo M (2010). A longitudinal study of sleep disorders in early-stage chronic kidney disease. J Ren Nutr.

[5] Sabbatini M, Pisani A, Crispo A (2008). Sleep quality in patients with chronic renal failure: A 3-year longitudinal study. Sleep Med.

[6] Stabouli S, Papadimitriou E, Printza N, Dotis J, Papachristou F (2016). Sleep disorders in pediatric chronic kidney disease patients. Pediatr Nephrol.

[7] Kumar B, Tilea A, Gillespie BW (2010). Significance of self-reported sleep quality (SQ) in chronic kidney disease (CKD): The Renal Research Institute (RRI)-CKD study. Clin Nephrol.

[8] Zhang J, Wang C, Gong W (2014). Association between sleep quality and cardiovascular damage in pre-dialysis patients with chronic kidney disease. BMC Nephrol.

[9] Stevens PE, Levin A (2013). Evaluation and management of chronic kidney disease: Synopsis of the kidney disease: Improving global outcomes 2012 clinical practice guideline. Ann Intern Med.

[10] Ravens-Sieberer U, Bullinger M (1998). Assessing health-related quality of life in chronically ill children with the German KINDL: First psychometric and content analytical results. Qual Life Res.

[11] Eser E, Yüksel H, Baydur H (2008). The psychometric properties of the new Turkish generic health-related quality of life questionnaire for children (Kid-KINDL). Turk J Psychiatry.

[12] Owens JA, Spirito A, McGuinn M (2000). The Children's Sleep Habits Questionnaire (CSHQ): Psychometric properties of a survey instrument for school-aged children. Sleep.

[13] Kirsztajn GM, Filho NS, Draibe SA, Netto MV, Thomé FS, Souza E, Bastos MG (2014). [Fast reading of the KDIGO 2012: Guidelines for evaluation and management of chronic kidney disease in clinical practice]. J Bras Nefrol.

[14] Elorza CL, Santos Junior AD, Celeri EH (2023). Quality of life, depression and anxiety in children and adolescents with CKD and their primary caregivers. J Bras Nefrol.

[15] Davis ID, Greenbaum LA, Gipson D (2012). Prevalence of sleep disturbances in children and adolescents with chronic kidney disease. Pediatr Nephrol.

[16] Safarpour Y, Vaziri ND, Jabbari B (2023). Restless legs syndrome in chronic kidney disease - A systematic review. Tremor Other Hyperkinet Mov (N Y).

[17] Kang KT, Lin MT, Chen YC, Lee CH, Hsu WC, Chang RE (2022). Prevalence of sleep disorders in children with chronic kidney disease: A meta-analysis. Pediatr Nephrol.

[18] Ahdoot RS, Kalantar-Zadeh K, Burton JO, Lockwood MB (2022). Novel approach to unpleasant symptom clusters surrounding pruritus in patients with chronic kidney disease and on dialysis therapy. Curr Opin Nephrol Hypertens.

[19] Baek HS, Kang HG, Choi HJ (2017). Health-related quality of life of children with pre-dialysis chronic kidney disease. Pediatr Nephrol.

[20] Stabouli S, Gidaris D, Printza N (2019). Sleep disorders and executive function in children and adolescents with chronic kidney disease. Sleep Med.

[21] Gerson AC, Wentz A, Abraham AG (2010). Health-related quality of life of children with mild to moderate chronic kidney disease. Pediatrics.

[22] Natale P, Ruospo M, Saglimbene VM, Palmer SC, Strippoli GF (2019). Interventions for improving sleep quality in people with chronic kidney disease. Cochrane Database Syst Rev.

